# Crystal structure of 1-(5-amino-2*H*-tetra­zol-2-yl)-2-methyl­propan-2-ol

**DOI:** 10.1107/S2056989015023713

**Published:** 2015-12-16

**Authors:** Hyun Sik Park, Ji Yeon Ryu, Junseong Lee

**Affiliations:** a Department of Chemistry, Chonnam National University, Gwangju 500-757, Republic of Korea

**Keywords:** crystal structure, 5-amino­tetra­zole, 2-methyl­propan-2-ol, hydrogen bonding

## Abstract

The title compound, C_5_H_11_N_5_O, crystallized with two independent mol­ecules in the asymmetric unit. The two mol­ecules differ in the orientation of the 2-methyl­propan-2-ol unit, with the hy­droxy H atoms pointing in opposite directions. In the crystal, mol­ecules are linked *via* O—H⋯O and N—H⋯O hydrogen bonds, forming ribbons propagating along [10-1]. The ribbons are linked *via* N—H⋯N hydrogen bonds, forming a three-dimensional structure.

## Related literature   

For the crystal structure of 5-amino­tetra­zole monohydrate, see: Britts & Karle (1967 ▸); and for that of 5-amino­tetra­zole, see: Fujihisa *et al.* (2011 ▸). For the crystal structures of alkali salts of 5-amino­tetra­zole, see: Ernst *et al.* (2007 ▸). For the crystal structure of 5-azido-1*H*-tetra­zole, a highly explosive compound, see: Stierstorfer *et al.* (2008 ▸). For some examples of the use of 5-amino­tetra­zole in the synthesis of metal–organic frameworks, see: Karaghiosoff *et al.* (2009 ▸); Liu *et al.* (2013 ▸).
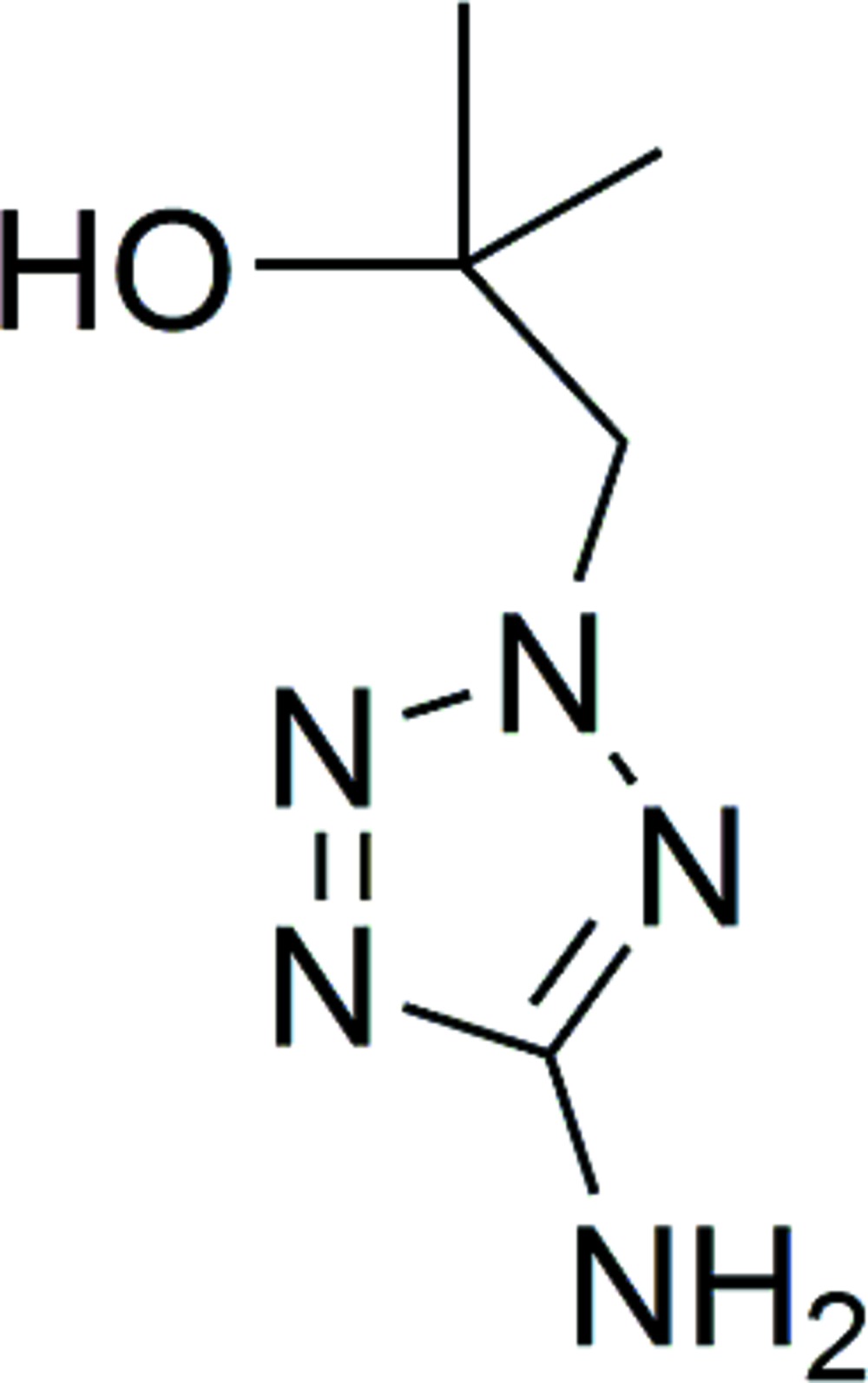


## Experimental   

### Crystal data  

C_5_H_11_N_5_O
*M* *_r_* = 157.19
Triclinic, 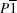

*a* = 8.2472 (19) Å
*b* = 9.731 (2) Å
*c* = 10.087 (2) Å
α = 90.30 (1)°
β = 96.228 (10)°
γ = 96.259 (10)°
*V* = 799.8 (3) Å^3^
*Z* = 4
Mo *K*α radiation
μ = 0.10 mm^−1^
*T* = 296 K
0.12 × 0.10 × 0.08 mm

### Data collection  

Bruker SMART 1K CCD diffractometer
Absorption correction: multi-scan (*SADABS*; Bruker, 2006 ▸) *T*_min_ = 0.90, *T*_max_ = 0.95
11190 measured reflections
2953 independent reflections
2148 reflections with *I* > 2σ(*I*)
*R*_int_ = 0.047

### Refinement  

*R*[*F* ^2^ > 2σ(*F* ^2^)] = 0.050
*wR*(*F* ^2^) = 0.122
*S* = 1.06
2953 reflections
227 parameters
H atoms treated by a mixture of independent and constrained refinement
Δρ_max_ = 0.17 e Å^−3^
Δρ_min_ = −0.19 e Å^−3^

### 

Data collection: *APEX2* (Bruker, 2006 ▸); cell refinement: *SAINT* (Bruker, 2006 ▸); data reduction: *SAINT*; program(s) used to solve structure: *SHELXS97* (Sheldrick, 2008 ▸); program(s) used to refine structure: *SHELXL2014* (Sheldrick, 2015 ▸); molecular graphics: *Mercury* (Macrae *et al.*, 2008 ▸) and *PLATON* (Spek, 2009 ▸); software used to prepare material for publication: *SHELXL2014* and *PLATON*.

## Supplementary Material

Crystal structure: contains datablock(s) I, New_Global_Publ_Block. DOI: 10.1107/S2056989015023713/su5257sup1.cif

Structure factors: contains datablock(s) I. DOI: 10.1107/S2056989015023713/su5257Isup2.hkl

Supporting information file. DOI: 10.1107/S2056989015023713/su5257Isup3.cml

**Fig. 1.**
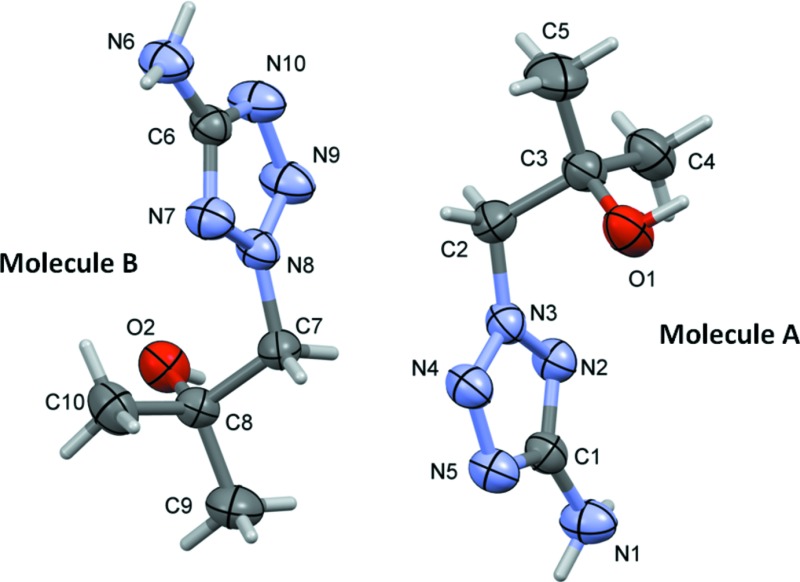
The molecular structure of the two independent molecules (A and B) of the title compound, with atom labelling. Displacement ellipsoids are drawn at the 50% probability level.

. DOI: 10.1107/S2056989015023713/su5257fig1.tif
The mol­ecular structure of the two independent mol­ecules (A and B) of the title compound, with atom labelling. Displacement ellipsoids are drawn at the 50% probability level.

**Fig. 2.**
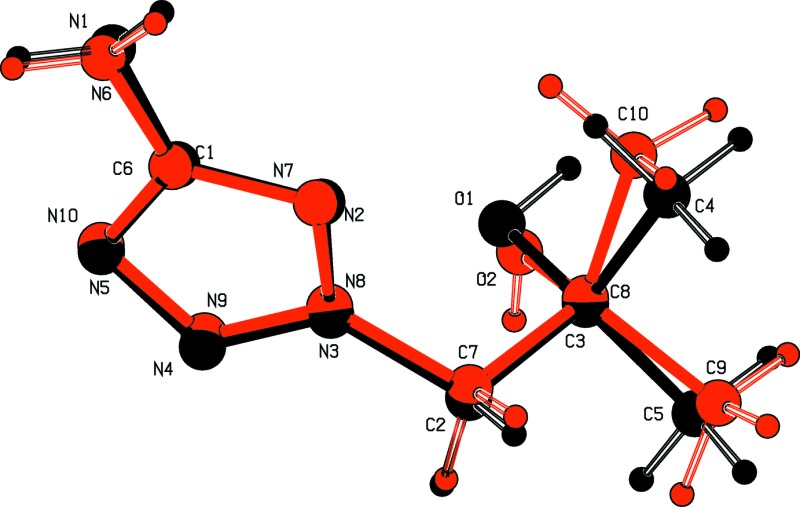
A view of the molecular overlap of molecules A (black) and B (red); calculated using the AutoMolfit routine in PLATON (Spek, 2009).

**Fig. 3.**
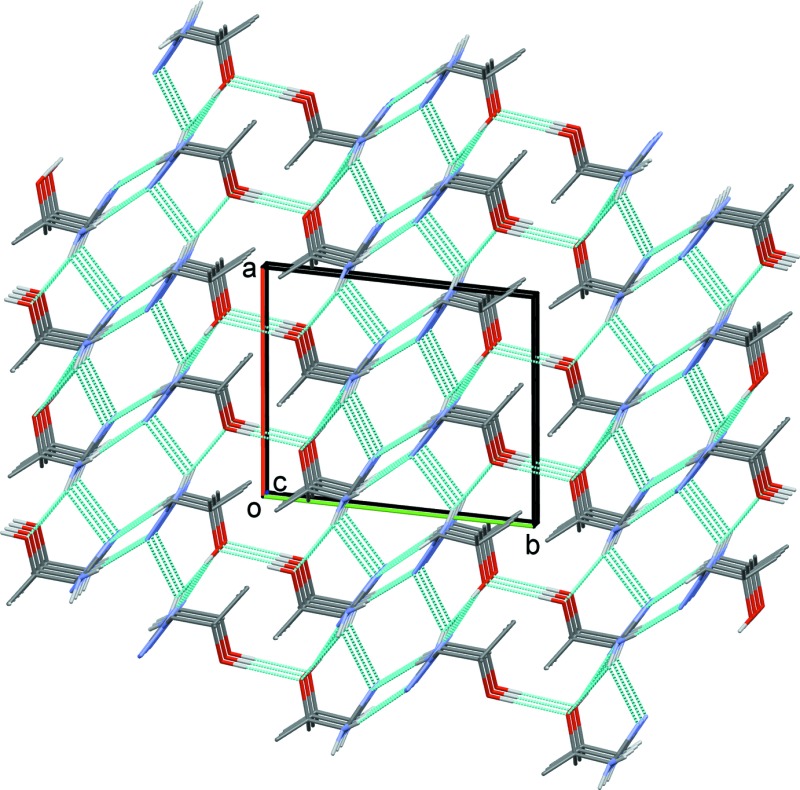
A view along the c axis of the crystal packing of the title compound. The hydrogen bonds are shown as dashed lines (see Table 1). H atoms not involved in hydrogen bonding have been omitted for clarity.

. DOI: 10.1107/S2056989015023713/su5257fig2.tif
A view of the mol­ecular overlap of mol­ecules A (black) and B (red); calculated using the AutoMolfit routine in PLATON (Spek, 2009).

c . DOI: 10.1107/S2056989015023713/su5257fig3.tif
A view along the *c* axis of the crystal packing of the title compound. The hydrogen bonds are shown as dashed lines (see Table 1). H atoms not involved in hydrogen bonding have been omitted for clarity.

CCDC reference: 1441577

Additional supporting information:  crystallographic information; 3D view; checkCIF report

## Figures and Tables

**Table 1:** Hydrogen-bond geometry (Å, °)

*D*—H⋯*A*	*D*—H	H⋯*A*	*D*⋯*A*	*D*—H⋯*A*
O1—H1*O*⋯O2^i^	0.91 (3)	2.04 (3)	2.946 (2)	171 (2)
N1—H1*A*⋯O2^ii^	0.92 (2)	2.53 (2)	3.243 (2)	135 (2)
N1—H1*A*⋯N9^ii^	0.92 (2)	2.58 (2)	3.287 (3)	134 (2)
N1—H1*B*⋯N10^iii^	0.84 (2)	2.24 (2)	3.082 (2)	173 (2)
O2—H2*O*⋯N2^ii^	0.82 (3)	2.14 (3)	2.930 (2)	162 (3)
N6—H6*A*⋯O1^iv^	0.93 (2)	2.22 (2)	3.114 (3)	161 (2)
N6—H6*B*⋯N5^v^	0.82 (2)	2.41 (2)	3.213 (2)	167 (2)

Symmetry codes: (i) 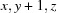; (ii) 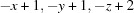; (iii) 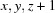; (iv) 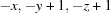; (v) 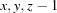.

## supplementary crystallographic information

### S1. Comments

Tetra­zole compounds are useful building blocks for the construction of high dimensional metal-organic frameworks, and they have provided various binding modes toward metal centers (Karaghiosoff *et al.*, 2009; Liu *et al.*, 2013). The title compound was easily prepared by the reaction of 5-amino­tetra­zole and *iso*-butyl­ene oxide, and introduces an hydroxyl group which we hope will be useful as an additional coordination center.

The title compound, Fig. 1, crystallized with two independent molecules(A and B) in the asymmetric unit. The two molecules differ in the orientation of the 2-methyl­propan-2-ol unit, with the hydroxyl H atoms pointing in opposite directions (Fig. 2).

In the crystal, molecules are linked *via* O—H···O and N—H···O hydrogen bonds (Table 1) forming ribbons propagating along direction [101]. The ribbons are linked *via* N—H···N hydrogen bonds forming a three-dimensional structure (Table 1 and Fig. 3).

### S2. Synthesis and crystallization

The title compound was synthesized by heating 5-amino­tetra­zole with an excess amount of *iso*-butyl­ene oxide, without solvent, at 333 K. Crystals were obtained on cooling the reaction mixture.

### S3. Refinement

Crystal data, data collection and structure refinement details are summarized in Table 2. The OH and NH_2_ H atoms were located in difference Fourier maps and freely refined. The C-bound H atoms were positioned geometrically and refined using a riding model: C—H = 0.96–0.97 Å with *U*_iso_(H) = 1.5*U*_eq_(*C*-methyl) and 1.2*U*_eq_(C) for other H atoms.

### Crystal data

**Table:**

C_5_H_11_N_5_O	*Z* = 4
*M**_r_* = 157.19	*F*(000) = 336
Triclinic, *P*1	*D*_x_ = 1.305 Mg m^−^^3^
*a* = 8.2472 (19) Å	Mo *K*α radiation, λ = 0.71073 Å
*b* = 9.731 (2) Å	Cell parameters from 4382 reflections
*c* = 10.087 (2) Å	θ = 2.0–29.9°
α = 90.30 (1)°	µ = 0.10 mm^−^^1^
β = 96.228 (10)°	*T* = 296 K
γ = 96.259 (10)°	Block, colourless
*V* = 799.8 (3) Å^3^	0.12 × 0.10 × 0.08 mm

### Data collection

**Table:**

Bruker SMART 1K CCD diffractometer	2953 independent reflections
Radiation source: fine-focus sealed tube	2148 reflections with *I* > 2σ(*I*)
Graphite monochromator	*R*_int_ = 0.047
profile data from ω scans	θ_max_ = 25.5°, θ_min_ = 2.5°
Absorption correction: multi-scan (*SADABS*; Bruker, 2006)	*h* = −9→9
*T*_min_ = 0.90, *T*_max_ = 0.95	*k* = −11→11
11190 measured reflections	*l* = −12→12

### Refinement

**Table:**

Refinement on *F*^2^	0 restraints
Least-squares matrix: full	Hydrogen site location: mixed
*R*[*F*^2^ > 2σ(*F*^2^)] = 0.050	H atoms treated by a mixture of independent and constrained refinement
*wR*(*F*^2^) = 0.122	*w* = 1/[σ^2^(*F*_o_^2^) + (0.0588*P*)^2^ + 0.018*P*] where *P* = (*F*_o_^2^ + 2*F*_c_^2^)/3
*S* = 1.06	(Δ/σ)_max_ < 0.001
2953 reflections	Δρ_max_ = 0.17 e Å^−^^3^
227 parameters	Δρ_min_ = −0.19 e Å^−^^3^

### Special details

**Table:**

Geometry. All e.s.d.'s (except the e.s.d. in the dihedral angle between two l.s. planes) are estimated using the full covariance matrix. The cell e.s.d.'s are taken into account individually in the estimation of e.s.d.'s in distances, angles and torsion angles; correlations between e.s.d.'s in cell parameters are only used when they are defined by crystal symmetry. An approximate (isotropic) treatment of cell e.s.d.'s is used for estimating e.s.d.'s involving l.s. planes.

### Fractional atomic coordinates and isotropic or equivalent isotropic displacement parameters (Å^2^)

**Table:**

	*x*	*y*	*z*	*U*_iso_*/*U*_eq_	
O1	0.28249 (16)	0.86314 (15)	0.81161 (14)	0.0536 (4)	
H1O	0.270 (3)	0.952 (3)	0.789 (3)	0.101 (9)*	
N1	0.4219 (2)	0.6570 (2)	1.28100 (17)	0.0530 (5)	
H1A	0.527 (3)	0.690 (2)	1.315 (2)	0.063 (7)*	
H1B	0.378 (2)	0.592 (2)	1.3251 (19)	0.048 (6)*	
N2	0.48661 (17)	0.69286 (15)	1.05833 (15)	0.0405 (4)	
N3	0.40258 (18)	0.64638 (14)	0.94250 (14)	0.0383 (4)	
N4	0.26829 (19)	0.56520 (15)	0.95690 (15)	0.0463 (4)	
N5	0.25930 (18)	0.55592 (16)	1.08764 (15)	0.0458 (4)	
C1	0.3924 (2)	0.63485 (18)	1.14668 (18)	0.0376 (4)	
C2	0.4501 (2)	0.68157 (18)	0.80986 (18)	0.0456 (5)	
H2A	0.3810	0.6223	0.7440	0.055*	
H2B	0.5624	0.6622	0.8062	0.055*	
C3	0.4370 (2)	0.83230 (18)	0.77215 (18)	0.0432 (5)	
C4	0.5765 (2)	0.9290 (2)	0.8434 (2)	0.0572 (6)	
H4A	0.5651	1.0223	0.8165	0.086*	
H4B	0.6794	0.9034	0.8208	0.086*	
H4C	0.5732	0.9226	0.9380	0.086*	
C5	0.4359 (3)	0.8413 (2)	0.6213 (2)	0.0730 (7)	
H5A	0.3447	0.7815	0.5786	0.110*	
H5B	0.5363	0.8133	0.5957	0.110*	
H5C	0.4260	0.9348	0.5945	0.110*	
O2	0.24050 (17)	0.15721 (15)	0.77069 (15)	0.0509 (4)	
H2O	0.301 (4)	0.198 (3)	0.831 (3)	0.131 (13)*	
N6	0.0533 (3)	0.3192 (2)	0.24056 (16)	0.0523 (5)	
H6A	−0.054 (3)	0.283 (2)	0.214 (2)	0.065 (7)*	
H6B	0.093 (2)	0.377 (2)	0.191 (2)	0.057 (7)*	
N7	0.00343 (18)	0.29922 (16)	0.46818 (14)	0.0436 (4)	
N8	0.09725 (18)	0.34753 (14)	0.57877 (14)	0.0382 (4)	
N9	0.23381 (19)	0.42091 (16)	0.55631 (15)	0.0505 (4)	
N10	0.23478 (19)	0.42088 (17)	0.42439 (15)	0.0516 (5)	
C6	0.0940 (2)	0.34662 (18)	0.37369 (17)	0.0377 (4)	
C7	0.0495 (2)	0.32626 (18)	0.71339 (17)	0.0416 (5)	
H7A	−0.0649	0.3416	0.7126	0.050*	
H7B	0.1138	0.3952	0.7729	0.050*	
C8	0.0714 (2)	0.18313 (18)	0.76967 (17)	0.0386 (4)	
C9	0.0260 (3)	0.1839 (2)	0.9121 (2)	0.0652 (7)	
H9A	0.0504	0.0992	0.9542	0.098*	
H9B	−0.0891	0.1923	0.9108	0.098*	
H9C	0.0881	0.2607	0.9610	0.098*	
C10	−0.0294 (3)	0.0686 (2)	0.6850 (2)	0.0573 (6)	
H10A	0.0133	0.0630	0.6005	0.086*	
H10B	−0.1416	0.0880	0.6710	0.086*	
H10C	−0.0237	−0.0178	0.7298	0.086*	

### Atomic displacement parameters (Å^2^)

**Table:**

	*U*^11^	*U*^22^	*U*^33^	*U*^12^	*U*^13^	*U*^23^
O1	0.0434 (8)	0.0474 (9)	0.0716 (10)	0.0081 (7)	0.0097 (7)	0.0127 (7)
N1	0.0503 (12)	0.0632 (12)	0.0421 (11)	−0.0055 (10)	0.0018 (9)	0.0068 (9)
N2	0.0397 (9)	0.0411 (9)	0.0389 (9)	0.0004 (7)	0.0010 (7)	0.0029 (7)
N3	0.0432 (9)	0.0329 (8)	0.0379 (9)	0.0030 (7)	0.0023 (7)	0.0032 (7)
N4	0.0500 (10)	0.0425 (9)	0.0436 (10)	−0.0031 (8)	0.0008 (7)	0.0055 (7)
N5	0.0436 (10)	0.0482 (10)	0.0436 (10)	−0.0011 (8)	0.0021 (7)	0.0076 (7)
C1	0.0356 (10)	0.0374 (10)	0.0395 (11)	0.0052 (8)	0.0015 (8)	0.0049 (8)
C2	0.0558 (12)	0.0436 (11)	0.0389 (11)	0.0073 (9)	0.0095 (9)	0.0002 (9)
C3	0.0446 (11)	0.0401 (11)	0.0454 (11)	0.0035 (9)	0.0083 (9)	0.0069 (9)
C4	0.0513 (13)	0.0492 (13)	0.0705 (15)	−0.0013 (10)	0.0098 (11)	0.0077 (11)
C5	0.0948 (19)	0.0761 (17)	0.0485 (13)	0.0069 (14)	0.0111 (13)	0.0190 (12)
O2	0.0433 (8)	0.0501 (9)	0.0586 (10)	0.0054 (7)	0.0015 (7)	0.0067 (7)
N6	0.0598 (12)	0.0577 (12)	0.0362 (10)	−0.0039 (10)	0.0007 (9)	0.0061 (8)
N7	0.0432 (9)	0.0492 (10)	0.0355 (9)	−0.0032 (7)	−0.0013 (7)	0.0030 (7)
N8	0.0410 (9)	0.0374 (9)	0.0347 (9)	0.0003 (7)	0.0010 (7)	0.0052 (7)
N9	0.0506 (10)	0.0563 (11)	0.0403 (10)	−0.0081 (8)	−0.0015 (8)	0.0093 (8)
N10	0.0489 (10)	0.0637 (11)	0.0390 (9)	−0.0062 (8)	0.0027 (8)	0.0102 (8)
C6	0.0413 (11)	0.0361 (10)	0.0357 (10)	0.0051 (8)	0.0030 (8)	0.0059 (8)
C7	0.0529 (12)	0.0379 (11)	0.0348 (10)	0.0059 (9)	0.0078 (9)	0.0018 (8)
C8	0.0396 (11)	0.0383 (11)	0.0380 (10)	0.0031 (8)	0.0056 (8)	0.0049 (8)
C9	0.0835 (17)	0.0633 (15)	0.0513 (13)	0.0076 (12)	0.0191 (12)	0.0159 (11)
C10	0.0582 (14)	0.0447 (12)	0.0648 (14)	−0.0055 (10)	−0.0005 (11)	0.0037 (10)

### Geometric parameters (Å, º)

**Table:**

O1—C3	1.437 (2)	O2—C8	1.443 (2)
O1—H1O	0.91 (3)	O2—H2O	0.82 (3)
N1—C1	1.362 (2)	N6—C6	1.366 (2)
N1—H1A	0.92 (2)	N6—H6A	0.93 (2)
N1—H1B	0.844 (19)	N6—H6B	0.82 (2)
N2—C1	1.333 (2)	N7—C6	1.328 (2)
N2—N3	1.342 (2)	N7—N8	1.339 (2)
N3—N4	1.310 (2)	N8—N9	1.308 (2)
N3—C2	1.466 (2)	N8—C7	1.464 (2)
N4—N5	1.332 (2)	N9—N10	1.332 (2)
N5—C1	1.349 (2)	N10—C6	1.347 (2)
C2—C3	1.529 (3)	C7—C8	1.528 (2)
C2—H2A	0.9700	C7—H7A	0.9700
C2—H2B	0.9700	C7—H7B	0.9700
C3—C4	1.518 (3)	C8—C10	1.515 (3)
C3—C5	1.524 (3)	C8—C9	1.524 (2)
C4—H4A	0.9600	C9—H9A	0.9600
C4—H4B	0.9600	C9—H9B	0.9600
C4—H4C	0.9600	C9—H9C	0.9600
C5—H5A	0.9600	C10—H10A	0.9600
C5—H5B	0.9600	C10—H10B	0.9600
C5—H5C	0.9600	C10—H10C	0.9600
			
C3—O1—H1O	107.4 (15)	C8—O2—H2O	113 (2)
C1—N1—H1A	117.3 (13)	C6—N6—H6A	116.9 (13)
C1—N1—H1B	113.0 (14)	C6—N6—H6B	114.9 (15)
H1A—N1—H1B	114.2 (18)	H6A—N6—H6B	115.5 (19)
C1—N2—N3	101.66 (14)	C6—N7—N8	101.56 (14)
N4—N3—N2	113.70 (14)	N9—N8—N7	114.12 (14)
N4—N3—C2	121.24 (15)	N9—N8—C7	122.32 (15)
N2—N3—C2	125.06 (14)	N7—N8—C7	123.51 (14)
N3—N4—N5	106.41 (14)	N8—N9—N10	105.90 (15)
N4—N5—C1	105.97 (13)	N9—N10—C6	106.18 (14)
N2—C1—N5	112.25 (16)	N7—C6—N10	112.23 (16)
N2—C1—N1	124.33 (17)	N7—C6—N6	124.23 (18)
N5—C1—N1	123.35 (16)	N10—C6—N6	123.49 (16)
N3—C2—C3	114.27 (14)	N8—C7—C8	114.86 (14)
N3—C2—H2A	108.7	N8—C7—H7A	108.6
C3—C2—H2A	108.7	C8—C7—H7A	108.6
N3—C2—H2B	108.7	N8—C7—H7B	108.6
C3—C2—H2B	108.7	C8—C7—H7B	108.6
H2A—C2—H2B	107.6	H7A—C7—H7B	107.5
O1—C3—C4	110.33 (16)	O2—C8—C10	106.22 (15)
O1—C3—C5	110.39 (16)	O2—C8—C9	109.55 (15)
C4—C3—C5	111.14 (16)	C10—C8—C9	112.03 (15)
O1—C3—C2	105.38 (14)	O2—C8—C7	109.61 (13)
C4—C3—C2	111.81 (16)	C10—C8—C7	112.28 (15)
C5—C3—C2	107.60 (16)	C9—C8—C7	107.14 (15)
C3—C4—H4A	109.5	C8—C9—H9A	109.5
C3—C4—H4B	109.5	C8—C9—H9B	109.5
H4A—C4—H4B	109.5	H9A—C9—H9B	109.5
C3—C4—H4C	109.5	C8—C9—H9C	109.5
H4A—C4—H4C	109.5	H9A—C9—H9C	109.5
H4B—C4—H4C	109.5	H9B—C9—H9C	109.5
C3—C5—H5A	109.5	C8—C10—H10A	109.5
C3—C5—H5B	109.5	C8—C10—H10B	109.5
H5A—C5—H5B	109.5	H10A—C10—H10B	109.5
C3—C5—H5C	109.5	C8—C10—H10C	109.5
H5A—C5—H5C	109.5	H10A—C10—H10C	109.5
H5B—C5—H5C	109.5	H10B—C10—H10C	109.5
			
C1—N2—N3—N4	−0.60 (19)	C6—N7—N8—N9	1.0 (2)
C1—N2—N3—C2	178.59 (15)	C6—N7—N8—C7	178.36 (15)
N2—N3—N4—N5	0.2 (2)	N7—N8—N9—N10	−1.1 (2)
C2—N3—N4—N5	−178.98 (14)	C7—N8—N9—N10	−178.47 (14)
N3—N4—N5—C1	0.23 (19)	N8—N9—N10—C6	0.6 (2)
N3—N2—C1—N5	0.74 (19)	N8—N7—C6—N10	−0.5 (2)
N3—N2—C1—N1	−176.40 (17)	N8—N7—C6—N6	177.00 (17)
N4—N5—C1—N2	−0.6 (2)	N9—N10—C6—N7	−0.1 (2)
N4—N5—C1—N1	176.53 (17)	N9—N10—C6—N6	−177.61 (17)
N4—N3—C2—C3	110.47 (19)	N9—N8—C7—C8	−104.44 (19)
N2—N3—C2—C3	−68.7 (2)	N7—N8—C7—C8	78.4 (2)
N3—C2—C3—O1	−44.3 (2)	N8—C7—C8—O2	57.7 (2)
N3—C2—C3—C4	75.5 (2)	N8—C7—C8—C10	−60.1 (2)
N3—C2—C3—C5	−162.14 (16)	N8—C7—C8—C9	176.52 (15)

### Hydrogen-bond geometry (Å, º)

**Table:**

*D*—H···*A*	*D*—H	H···*A*	*D*···*A*	*D*—H···*A*
O1—H1*O*···O2^i^	0.91 (3)	2.04 (3)	2.946 (2)	171 (2)
N1—H1*A*···O2^ii^	0.92 (2)	2.53 (2)	3.243 (2)	135 (2)
N1—H1*A*···N9^ii^	0.92 (2)	2.58 (2)	3.287 (3)	134 (2)
N1—H1*B*···N10^iii^	0.84 (2)	2.24 (2)	3.082 (2)	173 (2)
O2—H2*O*···N2^ii^	0.82 (3)	2.14 (3)	2.930 (2)	162 (3)
N6—H6*A*···O1^iv^	0.93 (2)	2.22 (2)	3.114 (3)	161 (2)
N6—H6*B*···N5^v^	0.82 (2)	2.41 (2)	3.213 (2)	167 (2)

Symmetry codes: (i) *x*, *y*+1, *z*; (ii) −*x*+1, −*y*+1, −*z*+2; (iii) *x*, *y*, *z*+1; (iv) −*x*, −*y*+1, −*z*+1; (v) *x*, *y*, *z*−1.
