# Supplementary figures and images for: Crystal structure of 1-(5-amino-2H-tetra­zol-2-yl)-2-methyl­propan-2-ol
Source: Acta Crystallogr E Crystallogr Commun. 2015 Dec 16;71(Pt 12):o1057–8. doi: 10.1107/S2056989015023713 (PMC4719977; doi:10.1107/S2056989015023713)

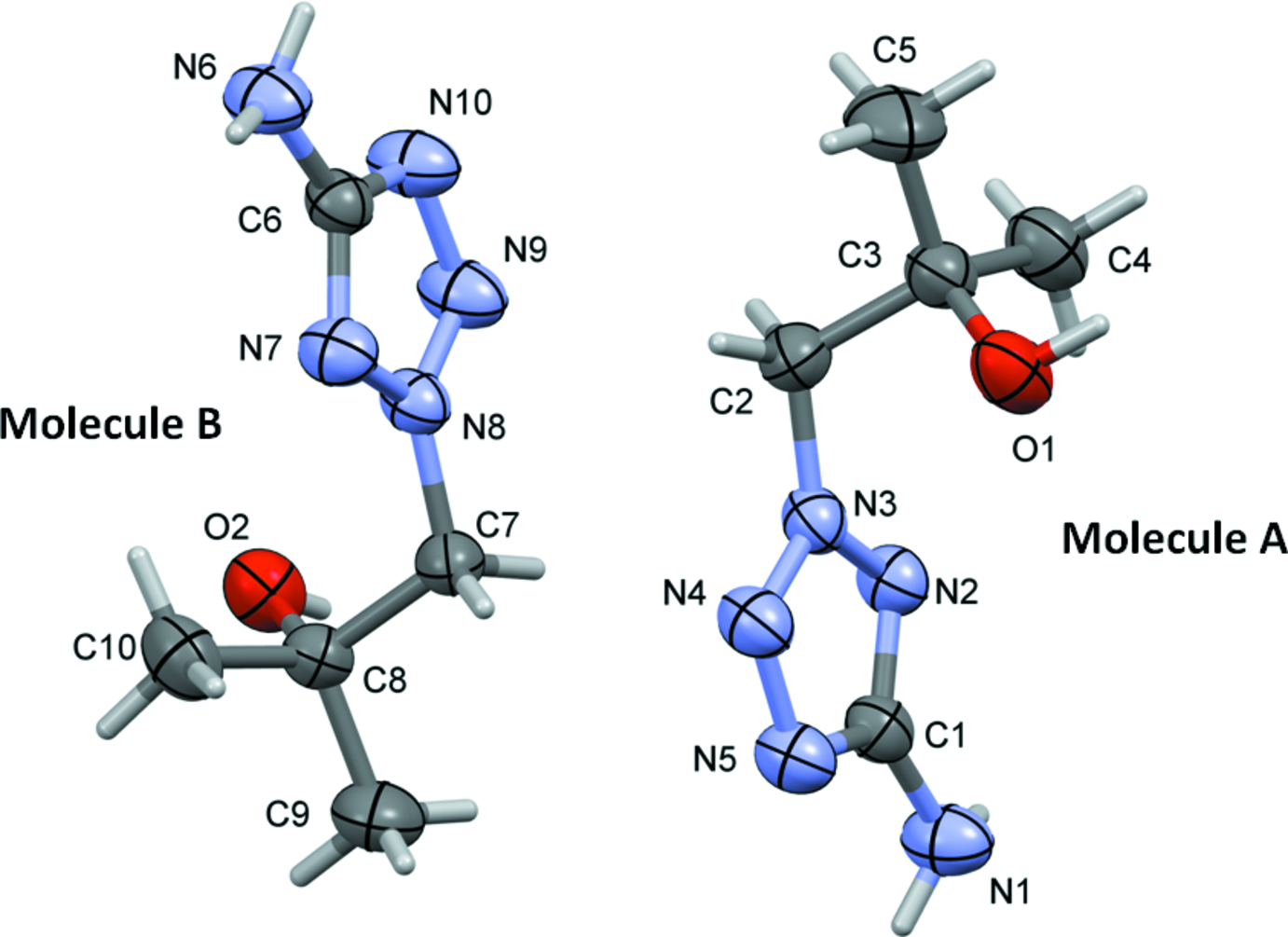

Supplement: Supplementary file 4 [file e-71-o1057-fig1.tif]

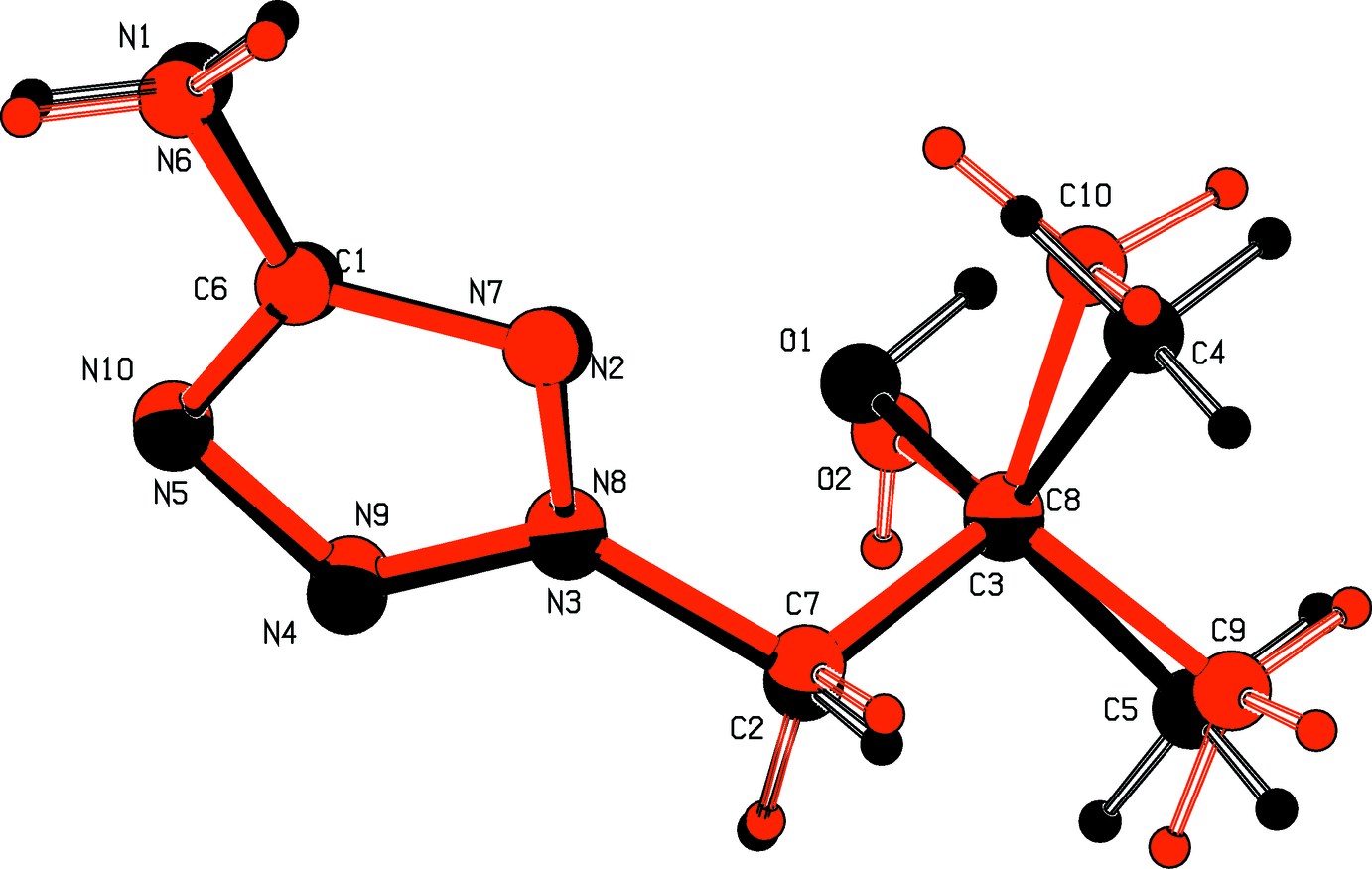

Supplement: Supplementary file 5 [file e-71-o1057-fig2.tif]

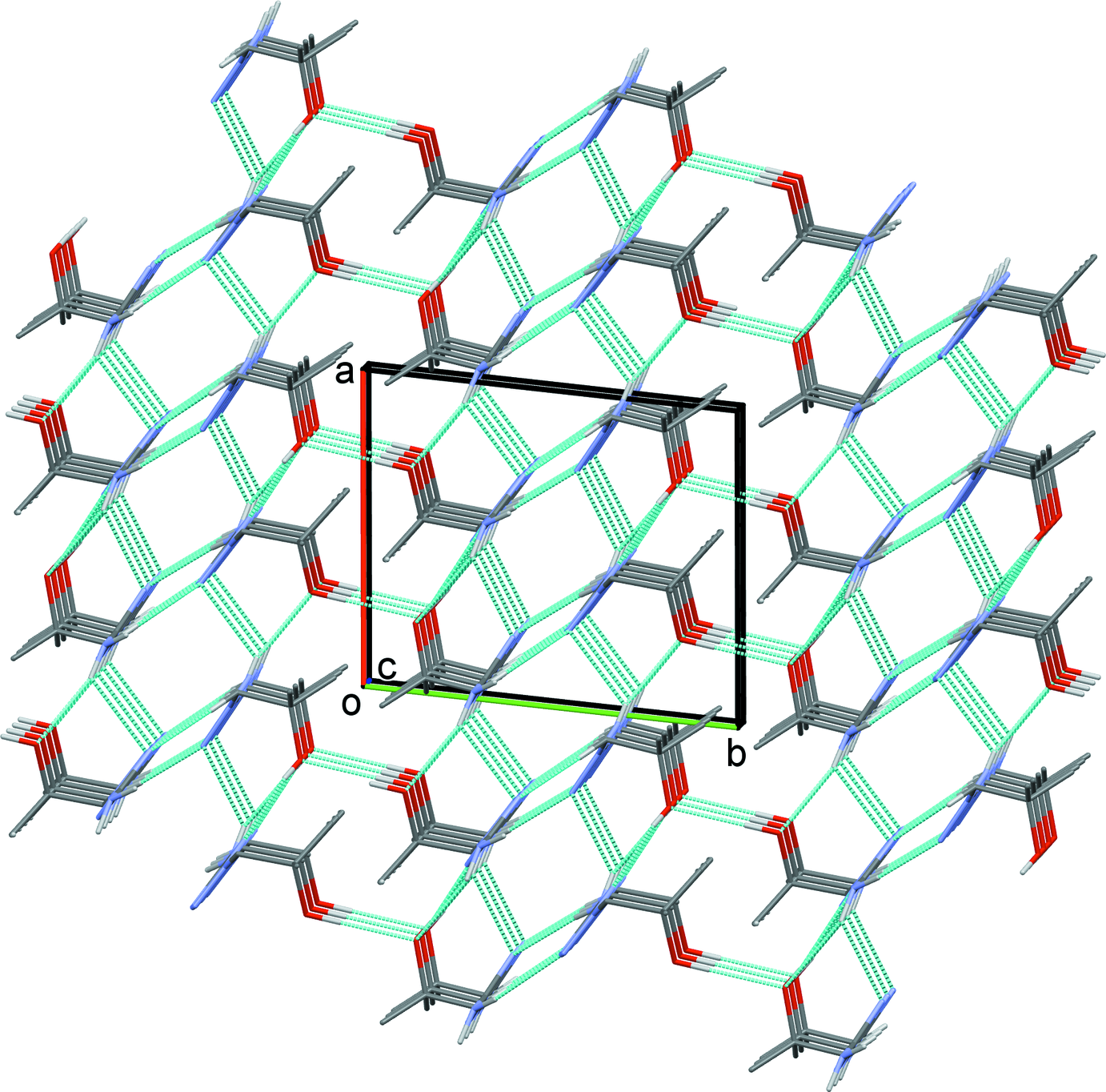

Supplement: Supplementary file 6 [file e-71-o1057-fig3.tif]
